# Exploring the role of SYK in respiratory *in vivo* models

**DOI:** 10.1186/1476-9255-10-S1-P11

**Published:** 2013-08-14

**Authors:** Michael Caniga, Janice D Woodhouse, Alan Wilhelm, Malgorzata A Gil, Yanlin Jia, Hongshi Yu, Stella Vincent, Richard Miller, Gissela Lieber, Xiomara Fernandez, Richard Chapman, Robbie McLeod, Lily Y Moy, Nancy Kelly, Emily Hickey, Michael A Crackower, Thomas Miller, William M Abraham, Milenko Cicmil

**Affiliations:** 1 Merck Research Laboratories, Boston, MA 02115, USA; 2 Merck Research Laboratories, Kenilworth, NJ 07033, USA; 3 Mount Sinai Medical Center, Miami Beach, FL 33140, USA

## Background

Spleen Tyrosine Kinase (SYK) is a key activator of signaling pathways downstream of multiple surface receptors implicated in asthma. SYK function has been extensively studied in Last cells downstream of the high-affinity IgE receptor (FcεR1). Most studies evaluating SYK function in preclinical models have relied on poorly selective compounds, anti-sense oligonucleotides, or SYK knockout mice. Here we describe the characterization of SYK mechanism in multiple in vivo model settings.

## Materials and methods

The effect of SYK inhibitor MRK-A on allergic airway responses was evaluated in IgE-mediated tracheal extravasation in rat, Brown Norway Ova rat models of allergic inflammation and the sheep inhaled ascaris allergen challenge model.

## Results

MRK-A dose-dependently blocked IgE-mediated tracheal extravasation in rat. In a Brown Norway rat ovalbumin-sensitized airway challenge model oral dosing of MRK-A led to a dose-dependent inhibition of airway inflammation. Intravenous dosing of MRK-A was able to significantly inhibit both early and late allergen- induced changes in airway resistance in an ascaris-sensitive sheep allergen challenge model as well as airway hyper responsiveness.

## Conclusions

Here we demonstrated that SYK mechanism plays a significant role in several in vivo allergen challenge models. This ranges from simple PK/PD mast cells driven models such as the IgE-mediated tracheal extravasation to the more complex and clinically relevant sheep inhaled allergen challenge model.

